# How Is Equity‐Based Co‐Creation Enacted in Health and Social Services? An Evaluation of Four Case Studies

**DOI:** 10.1111/hex.70647

**Published:** 2026-03-28

**Authors:** Samantha K. Micsinszki, Gillian Mulvale, Sandra Moll, Emma Bruce, Louise Murray‐Leung, Michelle Phoenix

**Affiliations:** ^1^ McMaster University Hamilton Canada; ^2^ CanChild Centre for Childhood Disability Research Hamilton Canada

**Keywords:** co‐creation, co‐design, equity, evaluation, health services, lived and living experience

## Abstract

**Introduction:**

Co‐creation processes are part of a movement to create change in collaboration with people with lived and living experience (PWLLE); in this paper we focus on experience with health and social services. Research co‐creation with PWLLE is increasing, however, processes tend to include people who are well connected in health and research environments, often excluding equity‐deserving groups (EDGs) who experience barriers to engagement in research and society more broadly (e.g., people with mental health conditions, immigrants, refugees, people who are unhoused).

**Methods:**

We conducted an evaluation to understand how equity‐based co‐creation (EqCC) is enacted in health and social services and what outcomes are produced. Informed by realist evaluation methods, we used multiple case study methodology to explore and describe how co‐creation was enacted in four sites that were actively conducting EqCC projects. The case study sites were engaging in projects on student mental health and well‐being; youth mental health transitions; individuals in critical care; and women experiencing complex long‐lasting homelessness. A variety of data sources were collected including interviews with 2‐3 key members of each project, organisational documents (e.g., meeting minutes, ethics approvals, reports), and observations. Data analysis included identifying the contexts, mechanisms, and outcomes, and causal configurations to develop a deductive coding scheme and codebook.

**Results:**

The causal configurations were found to be consistent with the data collected at the four sites: (1) Values (the culture and values that enable co‐creation); (2) Driving Issue (a community‐identified problem as the catalyst for the co‐creation to occur); (3) Organisational Infrastructure (the organisation's tangible infrastructure to support long‐term co‐creation, including funding, time, and training among others); and (4) Experiential Knowledge Network (people involved in the co‐creation, including the networks and partnerships created prior to, during, and as a result of the co‐creation).

**Conclusions:**

The causal configurations explored in this evaluation identify the key contexts, mechanisms and outcomes that support co‐creation with people from EDGs. These may inform inclusive co‐creation project design and the development of a middle range theory of equity‐based co‐creation.

**Patient or Public Contribution:**

This research was co‐designed and co‐produced by researchers and PWLLE who were members of the McMaster University Co‐Design Hub at the time of the project. The research questions guiding this work were informed through ongoing conversations with members of different communities affiliated with the McMaster University Co‐Design Hub. Co‐author LML identifies as a PWLLE and was instrumental in developing the research question, writing and discussing the causal configurations, and contextualising findings. We shared project updates at numerous team meetings (which included researchers and PWLLE) over the course of the research. Insights and feedback were sought at these meetings which helped to contextualise the work and understand the topic from multiple lenses, including PWLLE.

**Abbreviations**
EBCD: experience‐based co‐design
EDG: equity‐deserving group
EqCC: equity‐based co‐creation
PWLLE: people with lived and living experience

## Patient or Public Contribution

This research was co‐designed and co‐produced by researchers and PWLLE who were members of the McMaster University Co‐Design Hub at the time of the project. The research questions guiding this work were informed through ongoing conversations with members of different communities affiliated with the McMaster University Co‐Design Hub. Co‐author LML identifies as a PWLLE and was instrumental in developing the research question, writing and discussing the causal configurations, and contextualising findings. We shared project updates at numerous team meetings (which included researchers and PWLLE) over the course of the research. Insights and feedback were sought at these meetings which helped to contextualise the work and understand the topic from multiple lenses, including PWLLE.

## 1 Introduction

### 1.1 Principles of Co‐Design and Equity‐Based Co‐Creation

Co‐creation processes (inclusive of terms like co‐design, co‐production etc.) are part of a movement to create change through collaboration with people who have lived and living experience (PWLLE). The term PWLLE broadly describes people who have learned about the topic of interest, for example a particular health or social service, through their own life experiences. Other commonly used terms include ‘service user’ or ‘experience expert’. Our team has adopted the language of experience expert since a Canadian survey of over 600 PWLLE found that the term ‘experience expert’ was noted by the majority of PWLLE to accurately convey and legitimise the knowledge and skills they had in contributing as partners in health research environments [1]. However, the term was nuanced; some people felt it provided external validation for their expertise (e.g., their ideas were used in manuscripts, policies, or presentations) whereas others rejected the term expert, as it was seen to fit those with technical training [1]. This dialogue raises critical questions about whose expertise is sought out, valued, and used to inform health and social services [2, 3].

While there is a growing movement in which service providers, policy makers, and researchers include experience experts in co‐creation efforts, there is little consensus on what term/s to use to describe their processes. The term ‘co‐design’ has become ubiquitous [4] but has been inconsistently applied in the literature, often used interchangeably with similar terms such as co‐production, patient/consumer engagement or involvement [5]. There is no clear boundary around what ‘counts’ as co‐creation [6, 7, 8]. In this paper we have used the term co‐creation to encompass collaborative approaches to service design and research in which knowledge from experience experts is prioritised in understanding the issue and collaborative, creative and generative approaches to transformation in health and social services and systems are adopted [9]. A global rise in the use of co‐creation has been reported in the design of health services, with the intention of providing the public with increased opportunities to shape research that is relevant to their interests, with higher changes for implementation, and increased research quality [10, 11]. While some positive outcomes of co‐creation initiatives are reported, such as increased self‐determination, shared control and empathy, and advocacy [11], there are few evaluations of coproduction and best practices are unknown [10]. As such, a ‘dark side’ of co‐production has been described and points to challenges such as increased time and cost with negative impacts on researchers and interest‐holders [10]. A primary concern with this perspective is that it ‘neglects the unique and vital perspectives of patients, service users and marginalised citizens, and how this may have a detrimental impact on efforts to enable co‐production with marginalised and disadvantage groups’ [12] (p. 2).

This concern is well founded as many experience experts involved co‐creation efforts tend to include people who hold more privilege and are well connected in health and research environments, often by participating as partners in multiple studies [13]. Therefore, there have been calls to apply a justice and equity lens to critically examine who tends to be excluded, and to improve the meaningful inclusion of populations who are noted to be structurally marginalised [14]. Populations who face systemic barriers in accessing and receiving healthcare and are under‐represented as co‐creation partners ‘include (but are not limited to) Indigenous people, immigrants, refugees, and newcomers, and people with lived experience of mental health conditions’ [14]. Given the diversity among populations that may be excluded from research engagement opportunities, it is useful to adopt an intersectional approach to critically reflect on who may be excluded, the particular set of systemic barriers that may be experienced by that group and how best to build practices of engagement that are culturally responsive (i.e., understanding and acknowledging cultural practices and differences) and trauma informed (i.e., understanding and acknowledging that which may cause further harm and re‐traumatisation) [14, 15]. Our team has used the term ‘equity‐deserving’ groups (EDGs) to forefront the systemic barriers that may be limiting their inclusion in health and social service design and posing barriers to service use and optimal outcomes.

### 1.2 The McMaster University Co‐Design Hub

The McMaster University Co‐Design Hub [16] (the Hub) started as a 3‐year (2019‐2022) initiative to facilitate partnership formation, advance methods of co‐creation with EDGs, and enable knowledge sharing. The Hub was created out of an identified need to address the complex inequities that are faced by EDGs, and an acknowledgement that this requires innovative research methodologies to mobilise change. The Hub includes inter‐disciplinary researchers, students, service providers, and experience experts. Co‐creation, as noted by the Hub members, has the potential to create transformative change in complex systems, such as health, housing, employment, education, justice, settlement, domestic violence and other services that collectively support health and wellbeing of EDGs [17].

In 2021, the Hub undertook a developmental evaluation [18] approach inspired by experience‐based co‐design (EBCD) [19] to articulate our team's theory of change (a visualisation of our vision and strategies for the vision to become a reality) related to equity‐based co‐creation (EqCC) [20]. With support from community interest‐holders – which we define as ‘groups with legitimate interests in the health issue under consideration’ [21] (p. 2) – we identified ‘a need for changes in the way services are designed, delivered, and evaluated to be inclusive of structurally vulnerable populations’ [20]. (p300) In 2022, the Hub hosted an international symposium that brought together 48 people, including trainees, academic experts, and experience experts, from nine countries, who had expertise in co‐creation with people from often excluded groups in diverse health and social services. The full series of events and outcomes are reported online [22] and in peer‐reviewed manuscripts, including a charter that aims to enable reflexive co‐creation efforts with EDGs [9] and a collective vision paper that illustrates key principles and practices to advance EqCC [23]. These events allowed for the deep exploration of people's experiences in EqCC and created a shared opportunity for reflection and collaboration. We did not, however, evaluate co‐creation projects; thus, this project was undertaken to examine how co‐creation is enacted in real‐time across diverse environments and EDGs and to examine the outcomes of these initiatives.

## 2 Methods

### 2.1 Ethics Approval

Institutional ethics approval (REB# 10650) was received from the Hamilton Integrated Research Ethics Board at the Faculty of Health Sciences at McMaster University.

### 2.2 Study Design

This research used a multiple case studies [24] approach, informed by realist evaluation methods, to compare how EqCC is enacted in four projects that varied by setting and population. Case study methodology is appropriate when understanding the context is critical to the study of the phenomenon and ‘how’ or ‘why’ questions are being asked, and allows for the study of emergent processes and outcomes [24]. Given the heterogeneity in EDGs and in health and social services, case study is an ideal methodology for richly capturing the diversity across populations and the service context.

Realist evaluation ‘aims to advance understanding of why these complex interventions work, as well as how, for whom, in what context and to what extent—and also to explain the many situations in which a programme fails to achieve the anticipated benefit’ [25]. Realist evaluations are theory driven and promote exploration of the context, mechanisms, and outcomes of the intervention. Given that co‐creation is not a complex intervention, but there is still a need to determine how these processes work in health and social services, our team applied realist evaluation methods in the analysis phase of this multiple case study. Pivotal to this approach is an exploration of the contextual conditions in which a phenomenon occurs and the mechanism of action under that context in which an outcome can be explained, creating context‐mechanism‐outcome configurations [26].

Contexts refers to conditions in which a phenomenon occurs, and includes events, people, settings, and the resources and networks that link people, events, and places [27]. Mechanisms are the underlying constructs or processes that are context bound, can be activated at different system levels, and triggered at different timepoints [28, 29]. According to Astbury and Leeuw mechanisms are ‘usually hidden, sensitive to variation in context, and generate outcomes’ [30]. (p. 368) Outcomes are produced by mechanisms and may be planned or occur spontaneously, be positive or negative, and specific to the identified context and mechanism configuration [26, 27].

### 2.3 Sample and Recruitment

Purposeful sampling [31] was used to select four case study sites that were known to members of the research team to be conducting EqCC projects. The team selected local projects that reflected diversity among the EDGs and the type of health or social service provided. Case site leads were known by the research team and were approached to participate in this project if they were actively engaged in a co‐creation project (e.g., where service users were included at multiple points in the project design, implementation, and/or evaluation) regarding a health or social service with an EDG. Participants from each site purposefully included project leads and a range of people from diverse interest‐holder groups (e.g., experience experts, service providers, researchers).

### 2.4 Data Collection Methods

Multiple forms of data were collected at each case study site to allow for a holistic description of the context and object of study (e.g., co‐creation process and outcome). Theoretical saturation was used to determine the endpoint of data collection [32] (e.g., when enough information was thought to be collected to fully describe the context, mechanism and outcomes and the configuration between these elements).

Interviews were the primary data source. Online interviews were conducted via Zoom [33] with a variety of individuals at each site – including researchers and PWLLE – who participated in the co‐creation project at each site to understand context, mechanisms, and outcomes in the initial set of causal configurations. In this way, interviews were used to expand on the elements of the causal configurations by probing for specific details in each stage of the co‐creation project, including information about what went well and what could have been improved, and by identifying other elements of co‐creation that might not have yet been identified. Once the causal configurations were revised, organisational documents submitted by each case study site were reviewed and observation notes were explored for convergence and dissonance [34].

As secondary sources of data, documents and observations were used to gain depth and further understanding of the causal configurations, as well as validate what was heard in the interviews. Both publicly available and internal organisational documents were collected to provide further information about the context. Documents included organisational reports, journal publications, written materials about the co‐creation project (e.g., project newsletters, ethics review documents, previous project reports, letters of support), as well as documents generated through the co‐creation process (e.g., meeting minutes, arts‐based graphics).

Observations of team meetings or co‐creation sessions were conducted by SKM, EB, or a research assistant. The observations focused on the process of co‐creation, such as how individual experiences were elicited, how the team shared decision making, and what creative processes were used. An observational template was used to capture aspects of the co‐creation process consistently across sites, including who was invited to the event, interpersonal dynamics, and information shared. Due to the COVID‐19 pandemic, at the time of recruitment, the projects in our study were undergoing significant changes that impacted their study. Despite efforts to attend in‐person meetings and events, many were cancelled or postponed beyond our data collection period. An observation at one site could not be completed due to a delay in their scheduled event.

### 2.5 Data Analysis

To begin the data analysis, four members of our research team (SKM, MP, EB, and LML) identified the contexts, mechanisms, and outcomes and their causal configurations that described the process of co‐creation with people from EDGs. This primarily occurred through online discussions between four members of our team (SKM, MP, EB, and LML) from diverse disciplines (nursing, speech‐language pathology, occupational therapy, mental health, community development, system planning) and drawing from a combination of lived, clinical practice and research experience. This process occurred over 4 months and resulted in the development of a deductive coding scheme. The codebook that we created included each of the identified contexts, mechanism, and outcomes and allowed for new or emergent constructs to be coded, defined, and described.

All interviews were transcribed and de‐identified. Directed content analysis [35] was completed by SKM using NVivo 14 [36] and reviewed by MP using the deductive coding scheme and codebook. Memos were written to identify the specific context and mechanism of the causal configurations that resulted in outcomes at the individual, local system, and broader societal levels that reflected the causal configurations. Memos were also written in which the configurations between the context and mechanisms could be explored in relationship to the observed, reported and expected outcomes and challenges.

After all interviews were initially coded, SKM and MP analysed each interview. Key ideas were grouped together under each causal configuration and a summary of the similarities and key ideas was created for each causal configuration at each site. Particular attention was paid to where the causal configuration observed in the coded data diverged from the predicted configurations, these changes were recorded in memos and used to inform our final set of configurations as presented in the results. Specific examples were identified that robustly demonstrated the causal configurations. The summaries were then further combined to one summary for each causal configuration across all four sites. Throughout this process, SKM and MP met bi‐weekly to contextualise the data, revise and collapse the causal configurations, and ultimately refine the causal configurations that make up the final set of causal configurations. The causal configurations were then discussed with GM, SM, EB, and LML of the research team to allow for further revision and refinement.

## 3 Results

### 3.1 Description of Cases

Data was collected at four diverse case study sites. A description of each case study site and the data collected at each site is described in Table 1.

**Table 1:** Descriptions of the four case sites and the data collected at each site.

Case site	Description of case site	Data collected
**A**	A research programme evaluating a community‐based intervention for youth mental health transitions.	3 Interviews 16 documents 1 observation
**B**	A hospital‐based project to improve research consent processes for individuals who are in intensive care.	3 interviews 17 documents 1 observation
**C**	An arts‐based research project aimed at understanding the housing needs of women experiencing complex long‐lasting homelessness and reimagining housing support.	3 interviews 10 documents 0 observations
**D**	A recovery college mental health and wellbeing programme co‐created and led by students.	3 interviews 4 documents 1 observation
**All sites**		12 interviews 47 documents 3 observations

### 3.2 Description of the Causal Configurations

**Figure 1:**
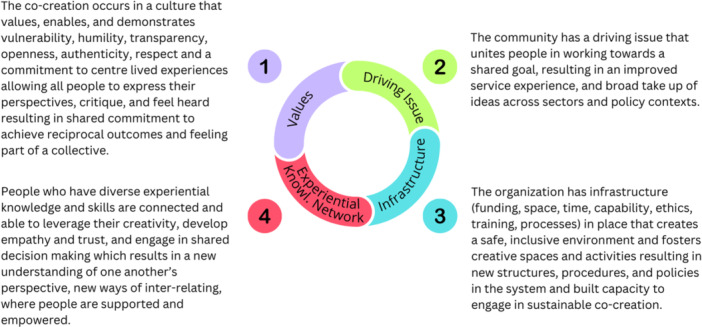
Visualisation of the final set of causal configurations.

There were four final causal configurations identified that together described how equity‐based co‐creation (EqCC) is enacted in health and social services and what outcomes are produced. These causal configurations were focused on values, the driving issue, organisational infrastructures, and an experiential knowledge network. Figure 1 illustrates how these causal configurations fit together to advance our understanding of EqCC. These causal configurations are presented and described below.

### 3.3 Causal Configuration 1: Values

**Final Causal Configuration for Values:** The co‐creation occurs in a culture that values, enables, and demonstrates vulnerability, humility, transparency, openness, authenticity, respect and a commitment to centre lived experiences allowing all people to express their perspectives, critique, and feel heard resulting in shared commitment to achieve reciprocal outcomes and feeling part of a collective.

All four case study sites described values that underpin their work. The values were described as foundational to doing EqCC and most important to the process as they set the tone and culture for the ways in which people worked together and what could be achieved. The values included trust, respect, authenticity, transparency, humility, and a constant need for growth and learning. Additionally, there was a consistent demonstration of the value and commitment to lived experience throughout the project. Through their various actions, sites showed their flexibility, responsiveness to needs, creativity, and open mindedness. The values were crucial to support the centreing of lived experiences, for example:

We can't make this work without developing really good relationships, rapport, all of those things to sustain those relationships over time, too, especially during periods of stress with COVID. So, I think stakeholder engagement is really our number one, and there's different ways in which we do that.

(Site A, Interview 1)

Lived experience was essential to the projects, and at two different sites it was cited as the ‘bread and butter’ of the work, where PWLLE were the driving force. Given the expressed values in how the work was conducted and the valuing of lived experiences, participants were able to express their perspectives on the project, critique the work, and feel heard. A critical part of the process was asking for feedback and creating opportunities to receive it, and being responsive to the feedback given. This required flexibility in the project methods (e.g., inviting partners to meet, delaying timelines due to COVID‐19 demands on community partners, providing multiple options for feedback). Embracing critique, and responding to it was often part of the shared decision‐making process. The following quote illustrates how Site A worked with youth advisors to receive feedback:

So if we're looking for feedback from our youth advisers…And then implementing that feedback and then demonstrating where the changes were made is part of our process. That's really important. Giving people different ways to provide feedback. So creating space in our meetings for people to give feedback but then also inviting separate feedback over email or whatever that might look like, given that people's availability changes.

(Site A, Interview 2)

Values of vulnerability, humility, transparency, openness, authenticity, respect, and commitment to centreing lived experience were critical in creating the context where the project could achieve reciprocal benefits, both for the community and for the partners from these communities who contributed to the projects. For example, partners stated that they were highly committed to and enjoyed connecting with other people who shared their vision. Individual outcomes experienced by the partners who engaged in co‐creation included co‐authorship, co‐presenting, learning about co‐creation, and feeling proud of their contributions and accomplishments. The researchers who engaged in co‐creation described learning more about their topic and also about how to do codesign.

The feedback that I'm getting from participants is that the process itself has been enjoyable. I know that they're enjoying it because they're sitting there way longer than they need to be. And at the end of it, they are like, “Oh, this was fun,’ or “This was good. I hope it leads to social change.’ I think that this population is often not really asked their perspectives on things…to be kind of participating in something that uses their own experience as legitimate knowledge and invites them to kind of imagine a better way is good.

(Site C, Interview 1)

Additionally, this created a positive space where people felt connected to each other. For example, at Site D, collective decisions were made through feedback and critique, but the students really led the process. They were able to talk things through, connect with one another, and feel pride and ownership of their work together, and this is reflected in the community and belonging they felt:

In the last session, students started sharing their social media pages with each other and people started becoming friends with each other, which I thought was really cool because that's not what we expected.

(Site D, Interview 1)

We observed a similar commitment to centre lived experiences during a team meeting at Site B by de‐centreing research experiences. In this meeting, the research leads on the project asked the student researcher to take the ‘Dr’ off their name on the beginning of a slide deck they were reviewing, reinforcing that ‘we are not the captains of the team…’. This helped to acknowledge that there is no hierarchy on the research team, that everyone's perspectives are valued and important.

### 3.4 Causal Configuration 2: Driving Issue

**Final Causal Configuration for Driving Issue:** The community has a driving issue that unites people in working towards a shared goal, resulting in an improved service experience, and broad take up of ideas across sectors and policy contexts.

All projects began in the community, outside of academia, where oftentimes it was patients or service users that identified the issue. This united teams in working towards a shared goal, where the problem was recognised by different parties who came together to work on it. For example, at Site A, the mental health navigation programme was well established in the community and the need for the programme had been identified by government reports. With a diverse team of community members, the project lead co‐wrote a paper describing the need for a more fulsome evaluation. The researchers and community members jointly identified the driving issue by discussing what successful project outcomes and their impact would be (e.g., influencing mental health policy and implementation of recommendations by partner organisations). At all case study sites, experience experts were integrated throughout the project from the beginning.

It's been really a co‐design project right from the beginning, right from the application for the initial grant. So, youth partners and family partners as well as obviously site‐specific folks were involved in supporting the initial grant development and writing letters of support for the activities of the study.

(Site A, Interview 2)

For two projects, the lead was already working in the community and had built connections and relationships. The current project was reported as an extension from previous clinical and research services and projects, which allowed the teams to develop a deep understanding of what the community needs and priorities were. Three projects reported some initial service experience improvements, for example ‘*after having our first research coordinator use the infographic…she thought that it really helped in terms of her own explanation of the study as well as the patient…their understanding*’ (Site B, Interview 1). However, we did not see enough evidence to suggest that a resolution of the issue would be possible, especially given that the projects were not complete at the time of data collection and the problems they addressed were complex and wide in scope. An example of the impossibility of resolving the issue, was recognised at Site C, noting the issue (homelessness) could not be resolved with a single project, even though it did have the possibility of creating some change locally.

I think that my work eventually will be shared in a way that could have some implications. I think that when organisations see the findings, I hope that they consider developing this kind of housing. It's very clear from my findings that all of the participants actually—well, all the participants self‐identified as wanting to live in this kind of housing. And this really is the group of participants who the city is having the most difficulty with supporting because they're unable to maintain the available housing options.

(Site C, Interview 3)

Despite not being able to fully resolve these complex issues, all projects had some initial success and evidence of some (broad) uptake of ideas across different sectors was currently happening or in process, such as buy‐in and interest, and some had already moved onto the next stages in funding.

I think that there's already international interest in this work. And further work in developing and testing the infographic was recently funded as part of the [larger programme funder] clinical trials project grants. So that's already been embedded into future clinical research.

(Site B, Interview 3)

### 3.5 Causal Configuration3: Organisational Infrastructure

**Final Causal Configuration for Organisational Infrastructure:** The organisation has infrastructure (funding, space, time, capability, ethics, training, processes) in place that creates a safe, inclusive environment, and fosters creative spaces and activities resulting in new structures, procedures, and policies in the system and built capacity to engage in sustainable co‐creation.

This causal configuration focused on the organisation's tangible infrastructure to support long‐term co‐creation, including funding, time, and training among others, and did not include people's skills/knowledge as infrastructure, which is elaborated on in causal configuration 4. Three case sites had been part of existing networks which were described as a valuable resource that allowed the team to build on established infrastructure such as resources, relationships, and continue momentum. For example, PWLLE at Site B were part of a large existing patient group in a larger trial where infrastructure to run the project was already in place.

So in terms of a financial perspective, the [larger study name] study also helps support this project. In terms of other resources, specific to this co‐design project, we haven't really had a need for any kind of physical resources because this team was already sort of existing in a sense and then adopted this study.

(Site B, Interview 1)

All sites described infrastructure supports and resources that aided them in enacting co‐creation. Funding, for example, was critical in hiring PWLLE to be a part of the team at all sites. Funding also allowed for opportunities to compensate participants, support co‐presentations, provide materials that improved accessibility (e.g., laptop for partner, art supplies, graphic designer). All four projects used flexible and creative methods tailored for things such as literacy, time available, preferences (e.g., interactive slides, photographs, maps, graphic designer). Site C shared documents that included the artwork created by participants in their co‐creation project – drawings, mind maps, and visualisations that were seen to encourage accessible and creative ways to engage:

And because my project is kind of an imagination—I'm asking people to imagine something that doesn't exist yet—so I think by engaging in these kinds of activities, I'm able to ground the conversation a little bit in terms of what exactly I'm hoping that they can think about and talk about.

(Site C, Interview 1)

At the case study sites, funding enabled participation and demonstrated care for the participants as it showed that their input was truly desired and valued. For example, in the following quote, funding was identified as a critical aspect of infrastructure that enabled the mechanism of creating a safe and inclusive environment:

It's actually the grant money [that] permits some of those things. And food and coffee and stuff, it's a respectful way, and it's a way to have conversations without like a PowerPoint presentation at the front of the room where we're all sitting and we're sharing food together, so. And I can tell you in interactions, it's like, ‘Really? We can order whatever we want off the menu?’ It was a small thing, but it was really important.

(Site A, Interview 3)

Infrastructure in the form of structured leadership roles helped to build capacity by allowing people to take on increased responsibility or additional roles on subsequent projects: ‘*I've gotten other jobs through working with [Researcher's Name] and stuff like that. I got a job with this organisation called [Community Organisation Name] where she helped me get a job with them, where we help homeless people*’ (Site C, Interview 2). Other participants, including both researchers and PWLLE described finding their own voice, increasing their confidence to engage in co‐creation, and forming relationships that were sustained post‐project. For example:

So it's been a learning process, but it's a learning process with respect, with honesty, with—now, as you could probably imagine, I'm not afraid to say anything at any point but it's also—and neither is [name1], but sometimes we may say that for other people or whatever—and not just me. It's just other people in the group, right? Make sure everybody has their say.

(Site B, Interview 2)

All projects noted that infrastructure was essential to the sustainability of the process. Sustainability was not part of the original causal configuration but was described frequently by participants. Sustainability was described in a variety of ways including the honoraria provided to engage, ability of team members to apply new skills to the next project and/or other environments, learning from the process of including family partners, and the consistency of people on the research team:

*So that's where building that rapport, having a consistent team‐‐ our team is small, but we're all kind of long haulers. And so I think having that consistency, having full‐time people to address all of this is super important*. (Site A, Interview 1)

While there was individual and project level capacity building, there was limited evidence to strongly support the creation of new permanent structures and policies within organisations that sustain or grow co‐creation.

### 3.6 Causal Configuration4: Experiential Knowledge Network

**Final Causal Configuration for Experiential Knowledge Network:** People who have diverse experiential knowledge and skills are connected and able to leverage their creativity, develop empathy and trust, and engage in shared decision making which results in a new understanding of one another's perspective, new ways of inter‐relating, where people are supported and empowered.

This causal configuration focused on people involved in the co‐creation, including the networks and partnerships created prior to, during, and as a result of the co‐creation. All case study sites included people with diverse experiential knowledge, skills, and relationships. This included PWLLE and other partners such as researchers, service providers, and policy makers. Three sites had well established networks for interconnected interest‐holders and were connected to ongoing projects/networks of projects which allowed people to develop relationships with one another. For example, at Site C, the project lead had strong community partnerships which made it possible to complete the work. As a previous frontline worker, the project lead was deeply connected to the community prior to engaging in the co‐creation project which was foundational to the project:

I've worked in this field for about 10 years and when the women come on Zoom, the majority of them I do know because they've been accessing the shelter system for so long, and I've worked in a number of women‐serving organisations and so that kind of relationship piece with the people I think is really helpful as well. And yeah, I've got some knowledge and understanding of what kind of information the city, local service planning tables are after. And so I've kind of used that inside knowledge to design the workshops in a way that, hopefully, will elicit some information that's helpful…

(Site C, Interview 1)

For most cases, the people engaging in the project were able to leverage their creativity and relationships to support this process. At Site D, we observed facilitators use an ice breaker activity to allow each participant to speak and used facilitation strategies to offer opportunities for people to participate in ways that were comfortable to them (i.e., told they can have their cameras on or off; can sit back, close their eyes, participate as they see fit; and reassured participants stating *whatever comes to mind, let's write it down, and then we can work out later where it specifically fits*). During the interview, a participant from Site D shared that a graphic designer was also used to support decision making by connecting with co‐creation participants and eliciting feedback in creative ways, such as through imaginative questions. Various non‐monetary supports, like personal check‐ins between researchers and partners, were used to create a safe and inclusive environment and each site had multiple examples of how they did this. Site A provided an example of how their team was responsive to the needs of the team through informal check‐ins:

There's never a meeting that goes by where we don't ask how everyone is doing, so that we can understand from their perspective where they're coming from, and what may work or not work for them. If we have to cancel a meeting because they're like, ‘Well, this is too stressful a time for everyone right now. Maybe it's not necessary for us to have this biweekly meeting at the moment.’ We'll re‐engage with them when things have calmed down a little bit.

(Site A, Interview 1)

At all sites, time was needed to develop relationships, empathy, and trust. Working together for a long period of time helped to build trust and develop empathy. For example, at Site B, the project lead was a research trainee and found it challenging to establish relationships when joining an existing team but that evolved over time which led to new ways of inter‐relating:

It can be challenging to engage everyone on every kind of aspect of the study as we've gone along. I would say that's less of a barrier for me now, only because I've developed relationships with everyone on the study. So I'm less worried about if this person—like if I don't reach out to this person, they're not going to be offended. I think the biggest thing that I've learned has been kind of providing that opportunity for everyone to be engaged and learning that people will kind of tell you when they want to be engaged, when they feel that they can meaningfully be engaged.

(Site B, Interview 1)

Engaging in shared decision making was marked by multiple examples at all sites. Sites described the collaborative decision‐making processes that they engaged in and the consensus methods used. In all cases, it was clear that the projects were co‐created with people that were going to be impacted by the project most.

When it boils down to me is: nothing about us without us. And having a variety of perspectives valued and really breaking down those barriers between service provider and consumer. And recognising consumers have expertise that's just as valuable as people who are in professional positions and things like that. So, I think it's really a blending of perspectives and evaluating of different perspectives to create something that's really robust and meaningful and sustainable and it's really a blending, I would say, to not only inform, but to design, to develop, to deliver, to promote.

(Site D, Interview 1)

Having an established relationship supported shared decision making and this was especially illustrated at Site A where trust was a mechanism needed for decision making and to troubleshoot together. Key interest‐holders had monthly meetings, provided updates, got feedback, and could make decisions about the project. Project teams who were established seemed to have a high level of consideration for people's time and energy in decision making and they were able to determine how much context to provide and to give feedback on changes made.

At the sites, researchers and PWLLE learned about each other's perspectives and needs. Within all sites, there was evidence that the researchers learned about lived/living experiences and that PWLLE learned about researchers’ perspectives (timelines, ethics).

Well, I got to learn a lot more about what others need in housing. I never really thought about it before. All this stuff's new to me. So doing this project, I learned a lot on what others need and being compassionate towards what they need because I never really thought about—I thought people were just needy before. And now I have a different perspective on it. All these people actually really need this kind of stuff. It benefits them. I don't know. It taught me a lot.

(Site C, Interview 2)

At all case study sites, we saw that participants felt supported and empowered:

So I've mentored our community partners, our navigators in this project to present at conferences or to their peers developing their CV or their resume because we have now publications. They're now the experts on it. So I think there are tangible [benefits]. I believe one indication is that the same people you're partnering with or co‐designing continue to say yes. To me, that ongoing, continued engagement is, one would think that it's because they're enjoying it or they get something out of it or they see a positive outcome.

(Site A, Interview 3)

## 4 Discussion

The objective of this evaluation was to advance understanding of why EqCC works, how it works, for whom, in what context and to what extent. Overall, the study findings provided evidence‐based support for four foundational and interconnected causal configurations that are essential for EqCC, including the 1) shared values; 2) a community‐identified ‘driving’ issue(s); 3) organisational infrastructure; and 4) experiential knowledge networks. While we did not explicitly explore the relationship between the causal configurations, we did observe that the values causal configuration was of utmost importance to the participants and appeared to be foundational to their EqCC experiences. The text that follows will explain the main findings within the contexts, mechanisms, and outcomes.

In terms of context, the study findings highlighted the importance of establishing a shared culture with values that centre lived experience. Building from established trusting relationships may help to identify issues that are of priority to the community prior to imposing an outsider's agenda. Bevan and colleagues—who consulted 20 individuals from diverse groups to understand how to improve equity, diversity, and inclusion in patient partnerships in policy research—state that ‘building on trust, participants said that involving people from the start of a project is important because it represents more genuine involvement, where their input is valued at every stage’ [15] (p4) This aligns with our findings in which, established clinical and research relationships by an individual or through an existing network, allowed the project teams to identify project aims and outcomes that were timely and of value to the communities that engaged in the co‐creation projects.

Study findings further suggest that there is context in terms of essential infrastructure needed to create a safe, inclusive environment. This included practical resources of funding, space, time, and skills/capacity of facilitators and participants. For example, teams recognised the value in compensating people for their contributions. In reality, however, pragmatic realities of securing funds in the project planning phases and continuing payment after a grant ends are often identified as challenges in research [37]. These realities may compromise teams’ ability to engage in EqCC as it is strongly recommended ‘that community members should be provided with the information, resources, and technological tools they need to participate’ [34] and without these resources, people who are privileged by income, education, and social capital are more likely to participate. The importance of non‐monetary support, such as offering flexible and inclusive practices that honoured the lived experiences of all team members and proactively met their needs, was highlighted in driving outcomes where people felt valued and wanted to continue their involvement. This is consistent with compensation frameworks and guidelines in Canada [37].

The study findings on mechanisms provide insights into how and why EqCC works. Findings that were highlighted in this project include: a) an emphasis on centreing lived experiences, and allowing all people to express their perspectives and feel heard (Values); b) uniting people in working towards a shared goal (Driving Issue); c) building a safe, inclusive environment (Organisational Infrastructure); and d) fostering creativity, empathy and trust, as well as shared decision making (Experiential Knowledge Network). All of these mechanisms are grounded in the importance of relational work which can be facilitated when people take time to build relationships and have infrastructure to sustain individual connections and networks over time. Trust and relationship building have been identified as key components of a trauma‐informed approach to patient‐engagement in health research [14]. Relationship building in a trauma‐informed approach allows people to feel cared for and to feel as though they are a part of a community [14]. These elements were seen as both mechanisms and outcomes in our study, in which people described the development of friendships and peer relationships as an unanticipated positive outcome and one that fostered their ability to work together. Building relationships and establishing trust may be particularly challenging when working with communities who have experienced ongoing or historical harms [9, 15, 38, 39]. In these circumstances, preparatory work and empathetic listening may be needed to understand the harms that exist within the community, identify and acknowledge our own biases, and to identify existing leaders in the community [9, 15].

Outcomes speak to the goals of the process, considering what works for whom and to what extent. Study findings noted outcomes at individual, relational, and system/policy levels. Individual level outcomes included feeling supported and empowered (Experiential Knowledge Network), and improved service experiences (Driving Issue) for PWLLE of illness, disability and structural discrimination. Relational outcomes included new understandings of different perspectives, new ways of relating to each other, feeling part of a collective and a shared commitment to change (Values and Organisational Infrastructure). These relational outcomes impacted all participants, from service users to researchers and service providers. Positive outcomes of increased empowerment, self‐determination and control and ownership have been reported when shared decision‐making and power‐sharing are included as throughout the codesign process [11].

When communities are provided with an opportunity to identify objectives that are meaningful to them, it increases their ownership of the project and likelihood of achieving results that are beneficial to the community. System or policy level outcomes included development of new structures, procedures, and policies, uptake of ideas across sectors and policy contexts (Values), and increased capacity to engage in sustainable co‐creation beyond the life of the project (Organisational Infrastructure). We noted that the intended outcome of ‘resolving’ issues was not necessarily met in the expected manner given the complexity of the issues being addressed (e.g., complex homelessness, informed consent in the context of the intensive care unit), but the process did provide the foundation for future system change.

Overall, the context mechanisms and outcomes identified in this project shed light on the cyclical and continuous nature of EqCC and sustainability was added as a key outcome in the final causal configuration for organisational infrastructure. The outcomes of one project often became the context in which a new project was created (e.g., fostering trusting relationships in which key priorities could be co‐identified) and/or other outcomes (e.g., shared power and control) became the mechanisms through which new co‐creation projects could achieve improvements in health and social services.

### 4.1 Strengths and Limitations

It should be noted that this research was conducted prospectively and the case studies in this project represented some diversity with respect to population (youth, women, hospital patients), and health service context (community care, hospital, education and housing). However, they were conducted in one geographic area in Ontario, Canada and are not representative of participants and foci in all EqCC projects. As such, the findings may not be directly transferable to other sectors and jurisdictions. Consistency of the findings across the diverse sites, however, speaks to the potential theoretical generalisability of the four causal configurations, to understand how co‐creation is enacted in other settings, particularly with respect to health and social services for communities who experience structural discrimination due to socioeconomic status, health status, and mental health. Additional research is needed to replicate findings in other settings across other EDGs.

This research also included multiple data sources, such as numerous internal and external documents related to processes of co‐creation. Direct observations of co‐creation activity (instead of retrospective reflections) are a strength. However, it should also be noted that this research represents a cross‐section of studies initiated at the beginning of the COVID‐19 pandemic, and therefore represents a unique temporal context characterised by the need to adapt methodologies in response to changing nature of engagement. For example, not being able to conduct research in‐person affected how co‐creation was enacted in the projects. It also impacted the nature of the data we were able to collect as a result (e.g., some in‐person observations were no longer possible). Future research is needed to examine the relevance of the causal configurations in other geographical and temporal contexts.

## 5 Conclusion

This evaluation was a unique and powerful opportunity to study EqCC in action within the real‐world context of community‐based research. The causal configurations helped to unpack the ‘black box’ of EqCC, considering the unique context for implementation, the key relational, functional, and structural mechanisms that drive outcomes, and reconceptualizing how to define success at an individual, group, and system level. By systematically comparing causal configurations across diverse studies, we have generated a broad framework that can be used to inform implementation and evaluation in future co‐design research with EDGs.

## Author Contributions

The development and evaluation of this work was done in partnership with members of the Equity‐Based Co‐Creation Centre (formerly the Co‐Design Hub for Vulnerable Populations). This research was conceptualised by all authors. Data collection was conducted by SKM and EB. Analysis was completed by SKM and MP with support from LML and EB. All authors provided feedback on this manuscript by discussion or in writing and have approved the final submission.

## Ethics Statement

Approval was granted by the Research Ethics Board at McMaster University (HiREB #10650; 3 September 2020). Informed consent was obtained from all individual participants included in the study.

## Conflicts of Interest

The authors have no conflicts or competing interests to disclose.

## Data Availability

The authors received no specific funding for this work.
